# Mating-Type Imputation (MTI) Provides an Efficient Tool for the Mating-Type Inference of Tetrapolar Fungi

**DOI:** 10.3390/jof12040287

**Published:** 2026-04-17

**Authors:** Zhenyang Yu, Yu Wang, Haixu Liu, Ruiheng Yang, Weijun Li, Huiyang Xiong, Yu Li, Yongping Fu, Shijun Xiao, Dapeng Bao

**Affiliations:** 1 College of Mycology, Jilin Agricultural University, Changchun 130118, China; 2 Institute of Edible Fungi, Shanghai Academy of Agricultural Sciences, Shanghai 201403, China; 3 School of Health Science and Engineering, University of Shanghai for Science and Technology, Shanghai 200093, China; 4 International Cooperation Research Center of China for New Germplasm Breeding of Edible Mushrooms, Jilin Agriculture University, Changchun 130118, China; 5 Jilin Province Key Laboratory of Fungal Phenomics, Jilin Agricultural University, Changchun 130118, China

**Keywords:** genetic diversity, mating-type, tetrapolar fungi, combinatorial pruning traversal

## Abstract

Mating-type identification is fundamental to studies of genetic diversity and genetic breeding in fungi, especially for tetrapolar basidiomycetes, whose mating types are determined by two multiallelic loci, A and B. Traditional mating-type identification of monokaryons relies on manual inference based on hybridization experiments; however, this process is highly complex, time-consuming, and error-prone when applied to large-scale studies. In this study, we isolated 30 monokaryons from protoplasts derived from 15 dikaryons of *Flammulina velutipes* and developed a software tool, Mating-Type Imputation (MTI), to automatically, rapidly, and accurately infer monokaryon mating types in tetrapolar fungi using a combinatorial pruning traversal algorithm. Using a compatibility matrix derived from 435 hybridization experiments involving these 30 monokaryons, MTI required only a few minutes to accurately infer the mating types of all monokaryons-a task that typically takes several days for manual inference by experienced investigators. Furthermore, MTI enabled us to investigate how false-positive and false-negative interactions influence mating-type inference results. Using a simulated compatibility matrix, we found that MTI could accurately detect potential false negatives in compatibility and successfully infer the true mating-type combinations even in the presence of limited false negatives; conversely, the tool was easily misled by any false positives, resulting in incorrect mating-type combinations. This indicates that false-positive records in hybridization experiments must be strictly eliminated during mating-type inference. In summary, MTI provides an efficient tool for inferring the mating types of tetrapolar fungi, offering technical support for mating-type studies of edible and medicinal fungi, and holds significant theoretical value and broad application potential in the fields of fungal genetic diversity and breeding research.

## 1. Introduction

Mating-type research is a prominent area of focus in mycological genetics and evolutionary biology, facilitating our understanding of fungal species isolation, genetic diversity, population structure, adaptive evolution, and crossbreeding [1,2]. Mating types determine the mode of sexual reproduction in fungi. Research indicates that mating types control the fusion of gametes and the formation of sexual spores, affecting genetic diversity and phenotypic variation within populations [3]. Furthermore, the highly diversified multiallelic mating-type loci in fungi provide valuable information for fungal taxonomy and germplasm tracing studies [2,4]. Previous studies have shown that the diversity of mating types reflects the population diversity of fungi and that mating-type alleles can serve as reliable molecular markers for clarifying taxonomic units and strain identification. From an applied perspective, understanding mating types holds significant practical value for fungal breeding and germplasm utilization. In the breeding of economically important fungi, such as edible and medicinal fungi, knowledge of parental mating types is fundamental for controlling the direction of crosses and improving the efficiency of genetic improvement [5]. Therefore, fungal mating-type research not only elucidates the fundamental biological principles of fungi at the genetic and evolutionary levels but also provides theoretical support for applied research in taxonomy and breeding programs [6,7,8].

The tetrapolar mating system is a prevalent, complex, and highly evolved sex recognition system in basidiomycetes [9,10]. This system operates via two mating-type loci, commonly referred to as the A locus and the B locus, which respectively control hyphal gamete recognition (A locus, encoding transcription factors such as HD1/HD2) and fusion behavior (B locus, encoding peptide pheromones and receptors) [11]. Most commercially cultivated edible and medicinal fungi, such as *Flammulina velutipes*, *Lentinula edodes*, *Ganoderma sichuanense*, and *Auricularia auricula*, exhibit a typical tetrapolar mating system [12,13]. Identification of the A and B mating-type loci of parents is essential for designing monokaryon crosses, enabling purposeful recombination of desirable traits and enhancing breeding efficiency [14,15]. Additionally, in agricultural and ecological systems, the prevalence of diseases caused by tetrapolar basidiomycetes (such as rusts and smuts) is closely related to their mating-type distribution [16,17,18]. Therefore, accurate mating-type identification is crucial for understanding disease transmission mechanisms and formulating control strategies [19,20].

The strain compatibility matrix deduced from pairwise monokaryon hybridization experiments provides the primary data for mating-type identification of tetrapolar basidiomycetes, but the manual mating-type inference process is complex, time-consuming, and error-prone [21]. Particularly as population sizes grow, the workload for manual mating-type inference increases exponentially. Although many studies on mating-type identification have been previously reported, primarily focusing on edible and medicinal mushrooms, a universal, efficient, and automated mating-type inference method based on strain compatibility matrices is still lacking for tetrapolar fungi [7,22,23]. Consequently, there is an urgent need in current fungal genetics research to develop such a mating-type inference tool using compatibility information from hybridization experiments for large-scale monokaryon populations. This will not only improve the efficiency and accuracy of mating-type identification but also provide strong technical support for research in fungal population genetics, evolutionary biology, and breeding [24,25].

Based on these considerations, this study developed a software tool, Mating-Type Imputation (MTI), to infer monokaryon mating types using monokaryon hybridization experiments for tetrapolar basidiomycetes. By taking advantage of the high rate of compatibility among monokaryons, we applied a combinatorial pruning traversal algorithm to improve the efficiency of mating-type inference for large-scale monokaryon hybridization experiments. MTI was validated in mating-type studies of *Flammulina velutipes* and used to investigate how errors in the strain compatibility matrix influence the results of mating-type inference. In summary, MTI and its application in this study not only provide a reliable tool for mating-type inference of tetrapolar basidiomycetes but also help to establish a paradigm for monokaryon mating-type studies in fungi in general.

## 2. Materials and Methods

### 2.1. Development and Application of Mating-Type Inference Method

#### 2.1.1. Mating-Type Inference (MTI) Algorithm

**Figure 1:**
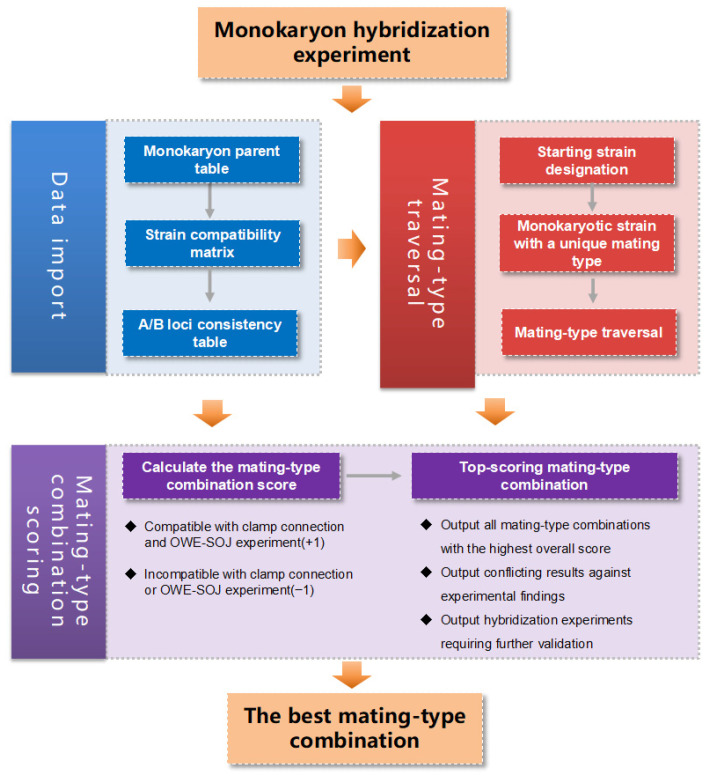
Analysis process of MTI mating-type inference software.

To achieve accurate mating-type inference, the MTI software v1 requires three inputs (Figure 1): first, the parental information of the monokaryon strains; second, the strain compatibility matrix derived from clamp connection observations in hybridization experiments [26,27]; and third, the loci consistency table derived from colony morphology observations of incompatible hybridization combinations in OWE-SOJ experiments [28].

The workflow of MTI for mating-type inference consists of the following steps (Figure 1 and Algorithm 1). Initially, users are required to provide two monokaryon strains with clearly distinct mating types as the starting strains; these are typically two monokaryon strains derived from a single dikaryotic parent, with their mating types designated as A_1_B_1_ and A_2_B_2_, respectively. Next, a sub-population of monokaryon strains exhibiting pairwise compatibility in the hybridizations is selected, and these monokaryons are labeled as A_3_B_3_, A_4_B_4_, and so on. Subsequently, the mating types of the remaining monokaryons, which show incompatibility in pairwise hybridizations, are traversed to generate all possible mating-type combinations. The algorithm evaluates each candidate assignment by calculating a total score based on its compatibility with all known strains: when the predicted mating outcome matches the observed result, the score increases by one score; when they conflict, the score decreases by one score. Incompatible matings are further evaluated against OWE-SOJ observations, with consistent outcomes adding one score and inconsistent outcomes subtracting one score. The scores of all mating-type combinations are then calculated to represent their overall consistency with the compatibility matrix (Figure 1). Finally, the mating-type combination with the highest score is identified as the optimal mating-type combination based on the hybridization experiments.
**Algorithm 1** Pseudo code of MTI for mating-type inference algorithm1:  Input: monokaryon parent table; strain compatibility matrix; A/B loci consistency table. 2:  Output: statistics on hybridization experiments; mating-type inference process; mating-types of monokaryon strains. 3:  Initialization: Graph; Pairing set; Result set. 4:  input.scan_error() 5:  for all monokaryotic strain: 6:   if monokaryotic.is assign(): 7:    result set.add(mating type) 8:   else: 9:    if monokaryotic.is one best() then: 10:      monokaryotic.assign and update graph() 11:     else: 12:      monokaryotic.unrecognized record() 13: for all unrecognized record: 14:  score = graph.traverse and calculate score() 15:  if score >= best score then: 16:      monokaryotic.assign and update graph() 17: for all graph path: 18:  if path score = total score then: 19:       result set.add(path.all type())

#### 2.1.2. Online Deployment of MTI

The MTI platform was developed using TypeScript 5.6.2 and Kotlin 2.0. The web frontend was constructed using a declarative, component-based technology model based on the Vue framework, which effectively reduces page refresh times, simplifies maintenance, and enhances the user experience [29]. The server backend was developed using the Ktor asynchronous framework, which offers advantages such as rapid application startup, low performance overhead, and high security, thereby significantly improving data analysis speeds [30,31]. For the visualization of mating types and sample data, the open-source OpenTiny HUICharts package 1.3.0 was employed. This package is based on ECharts and features advanced charting capabilities and rapid responsiveness [32]. For an optimal experience, we recommend accessing the website using JavaScript-supported browsers, such as Mozilla Firefox or Google Chrome.

### 2.2. Monokaryon Hybridization Experiment of Flammulina velutipes

#### 2.2.1. Strain Information

Fourteen wild strains and one cultivated strain of *Flammulina velutipes* used in this study were provided by the Edible Fungi Research Institute of the Shanghai Academy of Agricultural Sciences (Table 1). Using the technique of protoplast monokaryon isolation [33,34], 30 monokaryon strains were obtained.

#### 2.2.2. Experimental Instruments and Information

The instruments used in this study included an electronic balance (BS2202S; Sartorius, Göttingen, Germany), a shaker (ZHWY-100C; Shanghai Zhicheng Analysis Instrument Manufacturing Co., Ltd., Shanghai, China), a desktop constant temperature shaker (IS-RDD3; Crystal Industries, Lawrence, KS, USA), a desktop high-speed refrigerated centrifuge (Centrifuge 5810 R; Eppendorf, Hamburg, Germany), an upright microscope (Axio lab A1; ZEISS, Oberkochen, Germany), a clean bench (VS-840K-U; Suzhou Antai Air Technology Co., Ltd., Suzhou, China), a high-pressure steam sterilizer (SX-500; Tomy Digital Biology, Tokyo, Japan), and a constant temperature incubator (ZXSD-B1270; Shanghai Zhihang New Material Technology Co., Ltd., Shanghai, China).

#### 2.2.3. Culture Media Preparation

Potato Dextrose Agar (PDA) medium was prepared by dissolving 39 g of PDA powder in distilled water and adjusting the final volume to 1 L [35]. The solution was sterilized by autoclaving at 121 °C for 20 min.

Oak Wood Extract (OWE) medium was prepared as follows [28]: Briefly, 50 g of oak wood chips were suspended in distilled water, and the volume was adjusted to 1 L. The suspension was boiled for 60 min and subsequently filtered through a double layer of gauze. Separately, 20 g of agar was dissolved in distilled water, and the volume was adjusted to 1 L. A 200 mL aliquot of the oak wood filtrate and the entire 1 L agar solution were sterilized independently by autoclaving at 121 °C for 20 min. After cooling both components to approximately 50 °C, they were thoroughly mixed and poured into Petri dishes to solidify into plates.

Squeezed orange juice (SOJ) medium was prepared by first dissolving 20 g of agar in distilled water and adjusting the final volume to 1 L [28]. This agar solution and 200 mL of freshly squeezed orange juice were sterilized independently by autoclaving at 121 °C for 20 min. After cooling to approximately 50 °C, the sterilized agar solution and orange juice were thoroughly mixed and poured into Petri dishes to solidify into plates.

#### 2.2.4. Strain Compatibility Matrix Among Monokaryon Strains by the Hybridization Experiment

Mycelial plugs (5 mm × 5 mm) of the tested strains were inoculated onto Petri dishes (60 mm in diameter) containing 5 mL of PDA medium, with the plugs spaced 10–15 mm apart. The cultures were incubated at 25 °C for 10–12 days. Once the mycelia grew and contacted each other, the resulting colonies were examined for the presence of clamp connections using an upright microscope. If clamp connections were identified, the paired monokaryon strains were considered mating-type compatible; conversely, if no clamp connections were observed, the two strains were considered mating-type incompatible [36]. The strain compatibility matrix for the monokaryons was constructed based on these clamp connection observations.

#### 2.2.5. A/B Loci Consistency Table for Incompatible Monokaryon Pairs Using the OWE-SOJ Experiment

Monokaryon strains exhibiting incompatibility in single-cross experiments were inoculated onto PDA medium and incubated at 25 °C for 6 days. Subsequently, mycelial plugs (5 mm in diameter) from the edges of purified colonies of two mutually incompatible strains were extracted using a cork borer and inoculated onto OWE medium, with the plugs spaced 5 mm apart. After the mycelia of the paired strains began to contact, an agar strip (approximately 2 mm wide and 35 mm long) containing the two inoculated plugs was cut perpendicularly to the interface line and transferred to SOJ medium. The cultures were incubated at 25 °C for 4–6 days, and the colony phenotypes were recorded. Based on the results of mating-type analysis using the OWE-SOJ assay in *Lentinula edodes*, the A/B loci consistency table was determined according to colony characteristics [28]. An A=B≠ mating reaction occurs between strains with identical A factors but different B factors, forming a velvety colony along the agar strip without clamp connections. Conversely, an A≠B= mating reaction occurs between strains with identical B factors but different A factors, forming a sparse hyphal contact zone between the two colonies without clamp connections.

## 3. Results

### 3.1. Implementation of MTI for Mating-Type Inference

**Figure 2:**
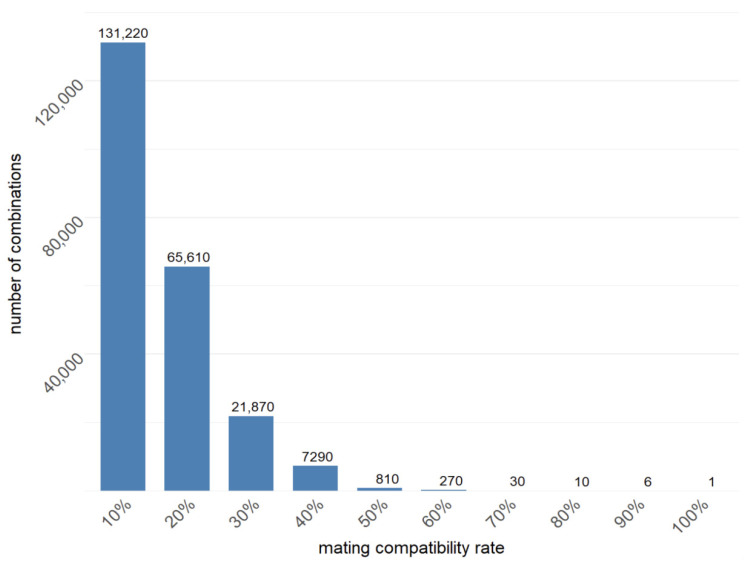
Number of traversing combinations with various mating compatibility rates. The number of mating-type combinations was listed on the bar.

The fundamental principle of mating-type inference in MTI is based on an exhaustive enumeration method. This method involves directly traversing all possible mating types among strains, generating the entire space of mating-type combinations. After calculating the scores for all combinations to represent their coherence with the strain compatibility matrix and the A/B loci consistency table, the combination with the highest score is recognized as the most likely set of monokaryon mating types. However, the number of combinations increases exponentially with the population size of monokaryons. For example, 10 monokaryons could lead to 1020 possible mating-type combinations, making the score computation for these combinations nearly impossible to complete within a reasonable time. To overcome this problem, MTI employs a combinatorial pruning traversal algorithm by taking advantage of the high mating compatibility rate among monokaryons. The basic logic of the algorithm is to fix monokaryons with clearly defined mating types to reduce the combination space among strains. To this end, the specific operation steps are as follows: first, a sub-population of strains that are pairwise compatible in the strain compatibility matrix is selected, since the mating types of these strains can be easily determined. Second, the exhaustive enumeration method is used to generate all possible mating-type combinations for the remaining monokaryons, thereby significantly reducing the complexity of score calculations. Using a population of 30 monokaryons with a 90% mating compatibility rate as an example, the combinatorial pruning traversal algorithm can reduce the 27,000 possible mating-type combinations to just six (Figure 2), significantly improving the efficiency of mating-type inference.

### 3.2. Application Platform Developed with MTI

**Figure 3:**
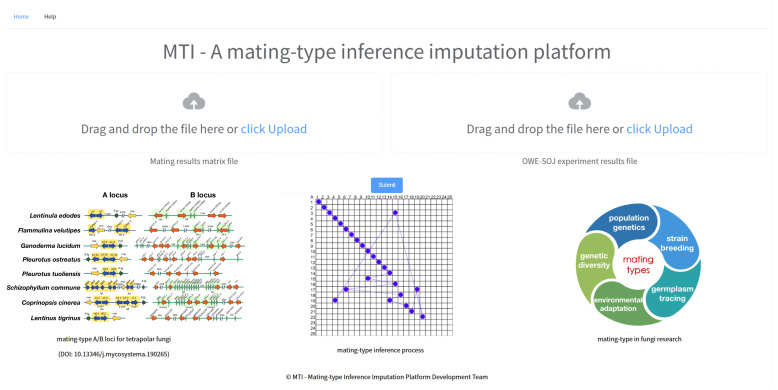
The snapshot of the online web platform with MTI. The online application needs the input of the monokaryon parent table, strain compatibility matrix, and A/B loci consistency table.

The source code for the MTI software has been uploaded to GitHub (https://github.com/bxx2004/MTI-web, accessed on 10 April 2026) and deployed online for public access (http://mti.myfungi.cn, accessed on 10 April 2026)). Sample data and user documentation for the software are provided in the GitHub repository. To facilitate the use of this software, we have developed a web platform for mating-type inference in tetrapolar basidiomycetes based on MTI. This platform consists of three modules: data input, statistics and analysis, and result display (Figure 3). The data input module facilitates user data uploads, the statistics and analysis module conducts analysis and inference based on the input data, and the result display module visualizes and prints the final mating-type results.

In the data input module, users can upload data tables with a single click. To ensure the accuracy and reproducibility of mating-type inference, MTI requires users to provide the following standardized input files (Table 2): (1) a monokaryon parent table, detailing the diploid parentage information of the monokaryon strains; (2) a strain compatibility matrix, containing the results of clamp connection observations from monokaryon hybridization experiments; and (3) an A/B loci consistency table, summarizing the results of colony trait observations from the OWE-SOJ experiments for all incompatible hybridizations. The MTI platform provides sample data and detailed file format specifications. After submitting the data, users can initiate mating-type inference by clicking the “Run” button.

The statistics and analysis module serves as the core processing component of the platform, conducting statistical analysis of the input data, executing the combinatorial pruning traversal algorithm for mating-type inference, and generating outputs for the subsequent results display module (Table 2).

The results display module focuses on visualizing the mating-type inference process and the resulting mating-type statistics (Table 2). The hybridization results statistics section summarizes basic information, such as the number of monokaryon strains, the number of hybridization experiments, and any missing hybridization combinations in the input data. The mating-type matrix is employed to visualize the mating-type inference process for the monokaryon strains. Finally, the mating-type result statistics section employs charts to illustrate the distribution of mating types across all monokaryon strains, along with the specific strain information associated with each mating type.

### 3.3. Mating-Type Inference for Flammulina velutipes Using MTI

To demonstrate the application of the software developed in this study, the mating types of *Flammulina velutipes* were inferred with MTI using the results of hybridization experiments. To this end, 30 monokaryon strains were subjected to pairwise hybridization experiments. Among the 435 possible pairwise combinations, 15 pairs derived from the same dikaryon were considered inherently compatible and were directly recorded as such without experimental testing, leaving 420 experimentally performed hybridizations. Based on microscopic examinations of clamp connections, 410 pairs showed compatibility, and 10 pairs showed incompatibility (Supplementary Table S1). The 10 incompatible hybridization pairs were subjected to the OWE-SOJ assay to probe the consistency of the A/B loci (Supplementary Table S2). In the OWE-SOJ experiment, the mating-type relationship between strains was determined based on colony characteristics, specifically whether the interaction was A=B≠ or A≠B=, thereby further clarifying the mating types of the strains under investigation. These observations were converted into a loci consistency table that records, for each pair of strains, whether their A loci are identical or different and whether their B loci are identical or different.

**Figure 4:**
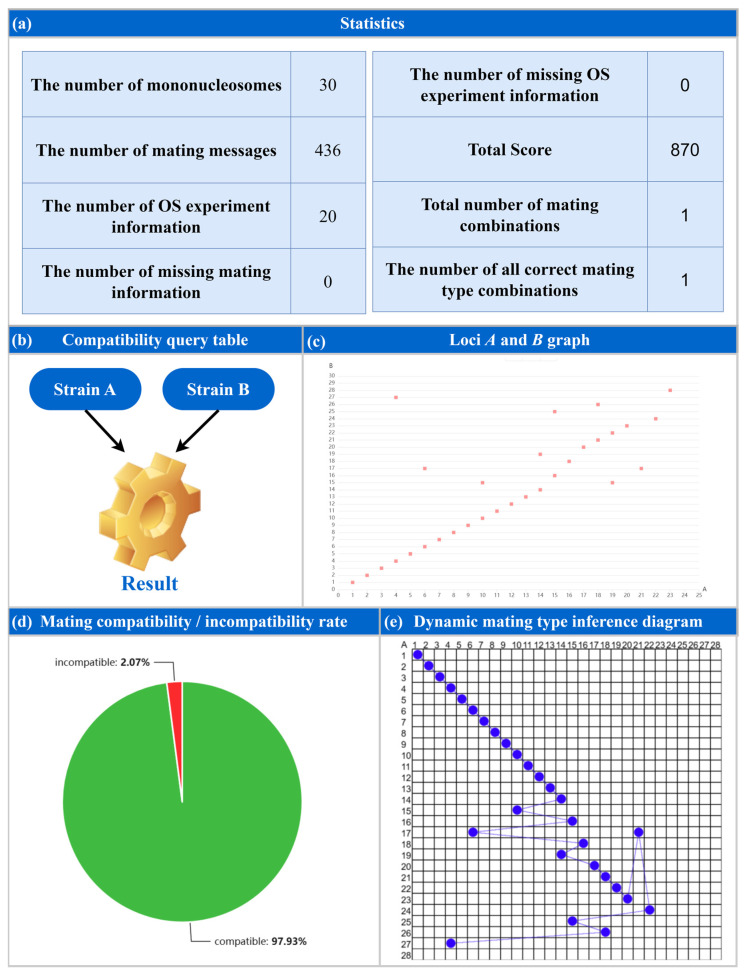
The results of mating-type inference using the MTI platform for *Flammulina velutipes*. (**a**) The statistics for input data and mating-type inference; (**b**) compatibility query function for strains; (**c**) the distribution of mating-types for all strains; (**d**) compatibility and incompatibility rate among strains; and (**e**) dynamic diagram for mating-type inference.

**Figure 5:**
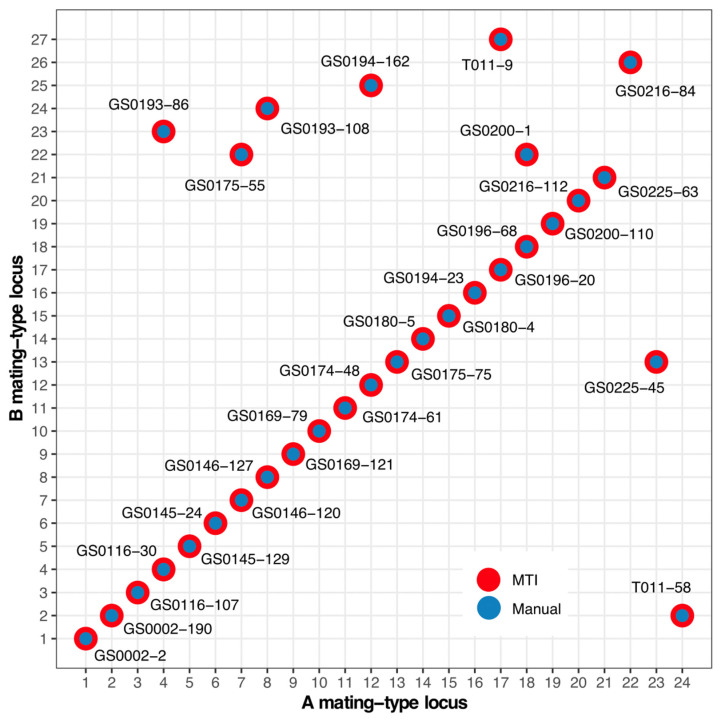
The validation of MTI by comparing with the manually inferred result.

Following the aforementioned experiments, the input file generated from the hybridization experiments involving the 30 *Flammulina velutipes* monokaryon strains was uploaded to the MTI web platform. Upon initiating the analysis, the system generated statistics for the input data and performed mating-type inference for the 30 monokaryon strains (Figure 4). Consequently, MTI inferred the optimal mating-type combination for the 30 monokaryon strains, achieving a maximum score of 870 that perfectly satisfied all hybridization experiment observations; this combination revealed 24 alleles at the A locus and 27 alleles at the B locus (Figure 5).

### 3.4. Manual Mating-Type Inference to Validate MTI Result

To validate the results of mating-type inference by MTI, we also performed a manual investigation to determine the mating types of the 30 monokaryon strains (Supplementary Materials). Based on the strain compatibility matrix and A/B loci consistency table from the hybridization and OWE-SOJ experiments (Supplementary Figure S1), the mating types of these 30 monokaryons were inferred manually, and the detailed process was recorded in Supplementary Materials. The entire manual inference process took approximately three days to complete. Ultimately, the manually inferred mating types of the 30 monokaryons were identical to the MTI results (Figure 5), thereby validating the accuracy of the software’s mating-type inference.

### 3.5. How Errors in Hybridization Experiments Influence Mating-Type Inference Results

Hybridization experiments provide the primary data for mating-type inference [37]; therefore, errors in the strain compatibility matrix pose potential challenges for accurate mating-type determination. However, how such errors in hybridization experiments influence mating-type inference remains largely unclear. MTI enabled us to investigate how input errors influence the results of mating-type inference using simulated data. False positives and false negatives are two common errors in hybridization experiments. To simulate hybridization experiments with errors, we randomly introduced one to five spurious compatible (false-positive) or incompatible (false-negative) records into the strain compatibility matrix of the 30 monokaryons. The mating types of the 30 monokaryons were inferred using these simulated datasets with MTI and compared with the true combinations.

**Figure 6:**
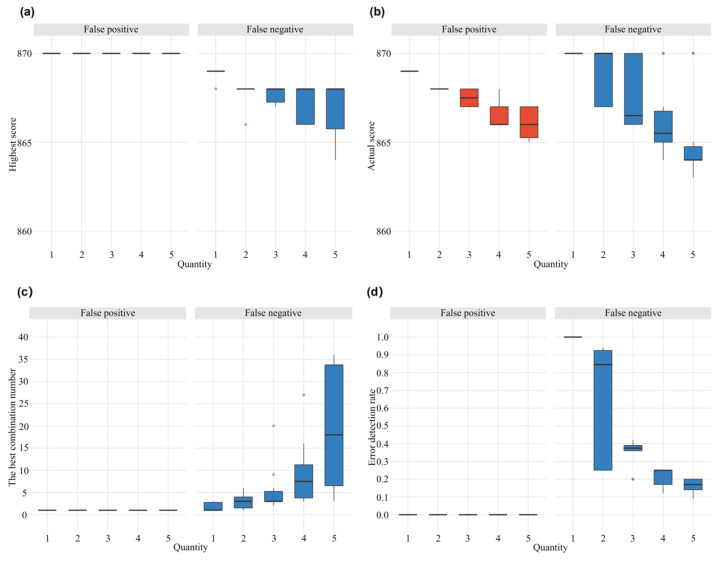
The impact of false-positives and false-negatives on mating-type inference. (**a**) The effect of random false-positives and false-negatives on the highest score. (**b**) The effect of random false-positives and false-negatives on the actual score. (**c**) The effect of random false-positives and false-negatives on the optimal combination number. (**d**) The effect of random false-positives and false-negatives on the accuracy of false-positive detection.

Our results revealed that false positives and false negatives exert distinctly different effects on mating-type inference. Any single false positive in the strain compatibility matrix could mislead MTI into deriving an incorrect optimal mating-type combination, even if that combination achieved a perfect score based on the flawed simulated dataset (Figure 6a,b). In contrast, false negatives in the simulated strain compatibility matrix reduced the overall mating-type combination score. Notably, if there was only one false negative, the resulting optimal mating-type combination remained identical to the true combination; however, when the number of false negatives exceeded one, multiple alternative optimal mating-type combinations were inferred, including the true one (Figure 6c).

Since errors in hybridization experiments can mislead mating-type inference, we subsequently investigated whether MTI could be used to detect false-positive or false-negative records in the strain compatibility matrix. Our analysis revealed that when the number of false negatives was small, especially in the case of a single false negative, MTI could accurately detect the error in the compatibility matrix, providing valuable information for experimental verification and tracing (Figure 6d). However, MTI failed to detect any false positives in our simulation analysis, because false positives consistently resulted in maximum-scoring mating-type combinations that perfectly satisfied the simulated strain compatibility matrix (Figure 6d). Our simulation analysis indicated that false positives exert a more substantial influence on mating-type inference and are significantly more difficult to trace. Therefore, greater emphasis must be placed on avoiding false-positive records during hybridization experiments, and the criteria for determining hybridization compatibility should be more stringent in practice. Unlike false negatives, which create inconsistencies that MTI can detect, false positives are fundamentally undetectable because there is no way to distinguish an erroneous positive from a true positive based solely on the compatibility data. If systematic false positives occur on a large scale, they can cause MTI to infer incorrect mating-type assignments. This highlights the critical importance of rigorous experimental quality control to minimize false positives.

## 4. Discussion

### 4.1. MTI as the First Mating-Type Inference Tool for Tetrapolar Fungi

The vast majority of medicinal and edible fungi are basidiomycetes and generally exhibit high mating-type diversity [38]. Mating-type identification serves as the foundation for studies of genetic diversity and breeding in these species [39]. Currently, the primary method for mating-type identification relies on monokaryon hybridization experiments [40]. Manual mating-type inference based on the strain compatibility matrix and A/B loci consistency is exceptionally labor-intensive and error-prone for large-scale monokaryon populations. This study established an automatic, rapid, and accurate method for inferring the mating types at the A/B loci of monokaryon strains in tetrapolar fungi using a strain compatibility matrix derived from clamp connection observations in hybridization experiments [37] and an A/B loci consistency table derived from colony phenotype records in OWE-SOJ experiments [4]. MTI was then further applied and validated using an *F. velutipes* monokaryon population. Specifically, the mating types of 30 monokaryon strains were rapidly and accurately inferred by MTI, and the results were identical to those obtained through manual inference. In summary, MTI represents the first mating-type inference tool for the majority of edible and medicinal fungi, as well as other tetrapolar fungi.

### 4.2. Main Features of MTI for Mating-Type Inference

To address the challenges of large-scale mating-type identification, the mating-type inference tool developed in this study for tetrapolar basidiomycetes demonstrates significant advantages in automation, speed, accuracy, and intelligence. First, MTI automates mating-type inference by eliminating the need for manual logical reasoning, thereby significantly lowering the technical threshold and difficulty associated with large-scale mating-type analyses. Second, MTI rapidly executes mating-type inference, especially in large populations. In the case of the 30 *F. velutipes* monokaryons, MTI required only two minutes to generate the results, whereas manual inference with identical input would take several days for experienced researchers. Third, MTI is highly accurate, as validated by the *F. velutipes* population. By relying on programmatic logic for mating-type inference, MTI avoids the subjective errors inherent to manual methods. Finally, MTI exhibits intelligent analytical capabilities; the software not only infers the optimal mating-type combination for monokaryon strains but also identifies potential errors in the strain compatibility matrix, providing valuable information for experimental design and data tracing.

### 4.3. Technical Scheme for Mating-Type Identification for Tetrapolar Fungi Using MTI

Based on the features and capabilities of MTI, we propose a standard technical protocol for monokaryon mating-type inference and mating-type library construction in tetrapolar fungi, which include the majority of edible and medicinal fungi. This protocol primarily consists of the following steps:

(1) Protoplast-derived monokaryons are isolated from dikaryotic parents to generate the monokaryon parent table;
(2) Pairwise hybridization experiments between monokaryons are conducted, and the strain compatibility matrix is constructed based on clamp connection observations;
(3) OWE-SOJ assays are conducted on incompatible monokaryon pairs to generate the A/B loci consistency table based on colony phenotype observations;
(4) Using the monokaryon parent table, the strain compatibility matrix, and the A/B loci consistency table, the mating types of all monokaryons are inferred via MTI software; these results are then used to establish a mating-type library for the species comprising representative strains;
(5) For a new monokaryon with an unknown mating type, hybridization experiments are conducted between the new monokaryon and the representative strains from the mating-type library;
(6) The mating type of the new monokaryon is determined via MTI software using the hybridization experiment results. If the new monokaryon possesses a mating type identical to one existing in the library, it is added to the strain list for that specific mating type. Conversely, if the new monokaryon exhibits a new mating type, the mating-type library is updated, and the new monokaryon is designated as the representative strain for this new mating type.

## 5. Conclusions

The MTI software provides an automatic, rapid, accurate, and intelligent solution for the mating-type inference of tetrapolar fungi, especially in large-scale populations. As the first tool specifically designed for mating-type inference, MTI not only simplifies the process of identifying mating types in tetrapolar fungi but also lays a solid foundation for a standardized research paradigm in mating-type library construction for these species. This plays an important role in studies of fungal genetic diversity, germplasm tracing, and strain breeding [41]. Therefore, MTI offers an efficient analytical tool for the fields of fungal genetics and diversity, holding significant theoretical value and broad application potential [42].

## Figures and Tables

**Table 1:** Information on the binucleate strains used in this study.

ID	Strain Number	Collection Site	Source
1	GS0002	Guan’e Gou Deadwood, Longnan, Gansu Province	Wild type
2	GS0116	Ping Shan Tang Dong Road, Yangzhou City, Jiangsu Province	Wild type
3	GS0145	Bengxing Village, Guihu Town, Chaozhou City, Guangdong Province	Wild type
4	GS0146	Dong Cao Wa, Guqiao Bridge, Fengtai County, Huainan City, Anhui Province	Wild type
5	GS0169	Campus of Huazhong Agricultural University, Wuhan City, Hubei Province	Wild type
6	GS0174	Huayuan Street, Xinjin District, Chengdu City, Sichuan Province	Wild type
7	GS0175	Yangxunqiao Street, Keqiao District, Shaoxing City, Zhejiang Province	Wild type
8	GS0180	Longtian Township, Yongxin County, Ji’an City, Jiangxi Province	Wild type
9	GS0193	Zhuzang Town, Zhijin County, Bijie City, Guizhou Province	Wild type
10	GS0194	Liufangtou, Gaoshan Town, Dongkou County, Shaoyang City, Hunan Province	Wild type
11	GS0196	Shaugulin, Qianjiang Town, Qianjiang District, Chongqing Municipality	Wild type
12	GS0200	Fufei Wubu, Fuzhou City, Fujian Province	Wild type
13	GS0216	Huancheng Road, Xiangcheng City, Zhoukou City, Henan Province	Wild type
14	GS0225	Lv Yinhu Street, Duyun City, Qiannan Buyei and Miao Autonomous Prefecture, Guizhou Province	Wild type
15	T011	Nagano Prefecture, Japan	Cultivation type

**Table 2:** Summary of input and output results for MTI.

Type	Number	Input/Output file	File Type	Information
inputs	1	Monokaryon parent table	Table	Monokaryon strain source information
	2	Strain compatibility matrix	Table	Compatibility of monokaryon strains from clamp connection observation in hybridization experiments
	3	A/B loci consistency table	Table	Compatibility of A/B loci from colony observation in OWE-SOJ experiments
outputs	4	Statistics on hybridization experiments	Table	Basic statistics on hybridization among monokaryon crosses
	5	Mating-type inference process	Image	Mating-type inference process of monokaryon strain
	6	Mating types of monokaryon strains	Table	All optimal mating-type combinations

## Data Availability

The software and data have been uploaded to GitHub (https://github.com/bxx2004/MTI-web, accessed on 10 April 2026) and are accessible via the MTI online platform (http://mti.myfungi.cn, accessed on 10 April 2026).

## Supplementary Materials

The following supporting information can be downloaded at: https://www.mdpi.com/article/10.3390/jof12040287/s1. Figure S1: OWE-SOJ experiment for 9 strains with undetermined mating-types; Table S1: hybridization experiment results among 9 strains with undetermined mating-types; Table S2: OWE-SOJ experiment results and mating-type analysis of 9 strains with undetermined mating-types.
